# Effects of substituting eggs for high-carbohydrate breakfast foods on the cardiometabolic risk-factor profile in adults at risk for type 2 diabetes mellitus

**DOI:** 10.1038/s41430-020-0599-2

**Published:** 2020-03-09

**Authors:** Kevin C. Maki, Orsolya M. Palacios, Melvyn W. Kramer, Rupal Trivedi, Mary R. Dicklin, Meredith L. Wilcox, Cathleen E. Maki

**Affiliations:** 1 Midwest Biomedical Research, Addison, IL USA; 2 MB Clinical Research, Boca Raton, FL USA; 3 Great Lakes Clinical Trials, Chicago, IL USA

**Keywords:** Pre-diabetes, Nutrition

## Abstract

**Objectives:**

To assess effects of egg-based versus non-egg, higher-carbohydrate (CHO) breakfast meals on cardiometabolic health markers in overweight or obese adults with prediabetes and/or metabolic syndrome.

**Methods:**

This randomized, crossover study included two 4-week dietary interventions, separated by a ≥4-week washout. Subjects incorporated into their habitual diets breakfast meals containing either 2 eggs/day for 6 days/week (Egg condition), or energy-matched, non-egg, higher-CHO-based foods (Non-Egg condition). Dietary intakes, insulin sensitivity, and other CHO metabolism indices, lipid biomarkers, high-sensitivity C-reactive protein, and blood pressures were measured.

**Results:**

Thirty men and women with mean age 54.1 ± 1.9 years and body mass index 31.9 ± 0.7 kg/m^2^ provided data. Neither diet condition significantly altered insulin sensitivity indices, but the homeostasis model assessment for insulin resistance was significantly (*p* = 0.028) higher after the Non-Egg vs. the Egg condition. Low-density lipoprotein cholesterol (LDL-C) was decreased from baseline (119 mg/dL) by 2.9 and 6.0% with Egg and Non-Egg breakfasts, respectively (*p* = 0.023). Systolic blood pressure was reduced from baseline (127 mm Hg) by 2.7 and 0.0% with Egg and Non-Egg, respectively (*p* = 0.018). Diet records indicated 149 kcal/day higher (*p* = 0.008) energy intake from non-study foods during the Egg condition; however, weight change from baseline did not differ between conditions.

**Conclusion:**

Compared with the baseline diet, consumption of 12 eggs/week for 4 weeks at breakfast was associated with less reduction in LDL-C, and more lowering of systolic blood pressure, than observed with non-egg-based, energy-matched, control foods higher in CHO.

## Introduction

According to the 2017 International Diabetes Federation Diabetes Atlas, there are 425 million people with diabetes in the world, and by 2045, there will be 629 million people worldwide with diagnosed and undiagnosed diabetes [1]. North America and the Caribbean have the highest regional prevalence of diabetes (15.4%). The rising prevalence of type 2 diabetes mellitus (T2D) is associated with higher levels of urbanization, aging populations, and unhealthy lifestyles, including obesity, inadequate physical activity, and higher intake of “unhealthy” foods [1]. This points to an urgent need to implement lifestyle recommendations to improve the cardiometabolic health profile [2]. In the United States, the Dietary Guidelines for Americans (DGA) 2015–2020 did not set a limit on egg consumption in a healthy diet [3]. However, results from some observational studies have raised concerns about egg consumption, and animal protein consumption in general, being associated with increased risk for T2D development. In a recent meta-analysis of prospective cohort studies, consumption of greater than 1 egg/day was associated with a 42% increased risk for incident T2D [4]. Egg consumption showed no association with T2D risk in another meta-analysis of prospective studies; however, a modest increase with consumption of ≥3 eggs/week was observed in US studies only [5]. In contrast, recent evidence from the Kuopio Ischemic Heart Disease Risk-Factor Study suggested that egg protein intake was associated with significantly reduced risk for T2D in Finnish men [6].

The independent effects of egg intake in these studies are difficult to determine, because egg consumption is associated with several other dietary and behavioral factors that could influence T2D risk [4]. Thus, the possibility of residual confounding cannot be ruled out. Furthermore, the paucity of randomized controlled trials evaluating the effects of egg intake on biomarkers of T2D risk, and the heterogeneity in interventions, study designs, and populations of these trials [7], limit the ability to draw conclusions about the influence of egg intake on the key determinants of glucose tolerance, i.e., insulin sensitivity and pancreatic beta-cell function.

In a randomized controlled crossover study in patients with controlled T2D, 1 whole egg/day at breakfast for 5 weeks reduced concentrations of the inflammatory marker tumor necrosis factor-α, compared with an oatmeal-based breakfast, without affecting glucose tolerance biomarkers [8]. A recently published study in adults with T2D showed that consumption of 2 eggs daily for 12 weeks did not significantly alter glycated hemoglobin levels, relative to a diet that excluded eggs, and improvements in anthropometric measures including body mass index (BMI), visceral fat, waist circumference, and percentage body fat were also observed [9]. Another examination of the consumption of 2 eggs/day, 6 days/week by individuals with prediabetes and T2D showed that both within the initial 3-month randomized controlled trial, and the follow-up trial of 9 months, egg intake did not adversely affect the lipid profile [10, 11]. Moreover, results from a randomized, double-blind, fully controlled feeding, crossover trial previously conducted in our laboratory showed that replacement of added sugars and refined starches with a combination of egg protein and unsaturated fatty acid (UFA) ingredients produced favorable changes in whole-body insulin sensitivity (net 24% increase) and lipoprotein-related variables [reduced triglycerides (TG) and very-low-density lipoprotein cholesterol and increased low-density lipoprotein (LDL) particle size] in men and women with hypertriglyceridemia [12].

Eggs contain cholesterol, but they are also a source of nutrients that may exert metabolically favorable effects, including UFA, high-quality protein, vitamin D, carotenoids, and choline [13]. Eggs are commonly consumed as part of breakfast meals. However, when eggs are not consumed, breakfast often contains other foods that are high in carbohydrate (CHO), particularly added sugars [14]. The present randomized, crossover study was designed to assess whether egg-based breakfast foods, providing 12 eggs per week, or higher-CHO, non-egg breakfast foods, for 4 weeks, would affect glucose homeostasis, lipoprotein-related variables, a marker of inflammation, and blood pressure in individuals at risk for T2D.

## Subjects and methods

### Design

This randomized, two-period, crossover trial was conducted in accordance with Good Clinical Practice Guidelines, the Declaration of Helsinki [15] and the United States 21 Code of Federal Regulations. An institutional review board (Aspire, Santee, CA) approved the protocol before initiation of the study, and subjects provided written informed consent before any study procedures were performed. The trial included two screening clinic visits, one baseline clinic visit, two 4-week dietary intervention periods with clinic visits after 3 and 4 weeks of each period, and a ≥4-week washout period between the intervention periods.

### Subjects

Men and women, 18–74 years of age, inclusive, each with a BMI of ≥25.0 kg/m^2^ (≥23.0 kg/m^2^ in Asian Americans), who met the criteria for having metabolic syndrome and/or prediabetes were eligible for the study [16, 17]. Subjects had to be otherwise healthy, have fasting TG levels <400 mg/dL, and be willing to consume only study-related foods and beverages at the breakfast meals during each intervention period. They also had to be willing to maintain their usual physical activity levels throughout the trial, and current smokers had to be willing to not change smoking habits during the study period. Potential subjects were excluded if they had atherosclerotic cardiovascular disease or a chronic inflammatory disease; pulmonary or endocrine (including type 1 diabetes and T2D) disease; a hepatic, renal, hematologic, immunologic, dermatologic, neurologic, psychiatric, or biliary disorder; or a recent history (prior 5 years) or the presence of cancer other than nonmelanoma skin cancer. Those who had experienced significant weight change (±4.5 kg in past 3 months), had extreme dietary habits, or who had a recent history or strong potential for drug or alcohol abuse were excluded. Subjects with uncontrolled hypertension (systolic blood pressure ≥160 mm Hg and/or diastolic blood pressure ≥100 mm Hg) or a known allergy, sensitivity, or intolerance (mild lactose intolerance was not exclusionary) to any study foods or their ingredients were also excluded. Eligible individuals were not permitted to have unstable use of any antihypertensive medication, or be actively taking antibiotics or dietary supplements or medications known to alter lipid (except for stable use of statins) or CHO metabolism, or be using weight loss drugs or programs. Subjects who were pregnant, planning to be pregnant during the study period, lactating, or of childbearing potential and unwilling to commit to the use of a medically approved form of contraception throughout the study period were also excluded. Last, individuals who had a condition the Investigator believed would interfere with his or her ability to provide informed consent, comply with the study protocol, or put the person at undue risk were excluded.

### Diets and procedures

During the first 4-week intervention period, eligible subjects were randomly assigned to one of the two dietary conditions [egg-based breakfast (Egg) or higher-CHO, non-egg-based breakfast (Non-Egg)]. Study products for each condition were provided using 3-day rotating menus. The egg-based breakfast meals (taco breakfast scramble, egg breakfast sandwich, and breakfast burrito) provided 2 whole eggs/day, 6 days/week (12 eggs/week), and the energy-matched breakfast meals without eggs contained higher-CHO foods. The egg-based breakfast meals were cooked by a caterer local to each of the two clinic sites (Chicago, IL and Boca Raton, FL), frozen and then dispensed to the subjects for their consumption at home. Subjects were given the frozen meals in a cooler bag with ice packs at the beginning and after 3 weeks of each 4-week intervention period. The subjects were instructed to place the frozen meals in their freezers as soon as they returned home, and were given instructions about how to reheat the meals for consumption. The non-egg study foods included ready to eat cereals with milk, waffles with syrup, granola bar, fruits, nuts, and cheese.

Mean energy and macronutrient contents of the study breakfast meals for both the Egg and Non-Egg conditions were ~554 kcal, 31–32% energy from fat and 10.5% energy from saturated fat. The main difference between the Egg and Non-Egg breakfast meals was a substitution of protein from eggs for sugar; 25 g of the 26 g difference between the Egg and Non-Egg breakfast meals was from sugar. Energy from protein was 25.8 and 12.1% of calories for the Egg and Non-Egg meals, respectively, and energy from CHO was 41.4 and 60.4% of calories, for Egg and Non-Egg meals, respectively (Supplementary Table 1). After the last day of the first diet condition, subjects were instructed to return to their habitual diets for the duration of the ≥4-week washout period, after which subjects crossed over to the other diet condition.

At baseline and during each intervention period, subjects received diet instruction on the incorporation of the study foods into their usual diets, with the aim of maintaining habitual energy intake. Subjects were also instructed to maintain their other lifestyle, including physical activity, habits. Baseline dietary intakes were assessed with 3-day diet records and analyzed using Food Processor^®^ Nutrition Analysis software (version 11.4, ESHA Research, Salem OR). The nutrient profiles of the Egg and Non-Egg breakfast meals, as well as the total daily dietary intakes during each intervention period obtained from 3-day diet records, were also analyzed. To assess compliance, subjects were instructed to complete a daily log in which they recorded study product consumption. Compliance was calculated by summing points and dividing by the number of days on treatment. If the study product was not consumed in its entirety, subjects documented whether 0, <50, or ≥50% had been consumed, and this was factored into the calculation of compliance. The Stanford 7-day Physical Activity Questionnaire was also completed at baseline and the end of each diet condition [18].

### Clinical and laboratory assessments

Clinic visit procedures, including body weight, waist circumference, and systolic and diastolic blood pressure measurements, were conducted at each clinic visit. Blood pressures were measured after the subject had been seated for 5 min. An automated device (Welch Allyn, Skaneateles Falls, NY or GE Dinamap V100, General Electric, Milwaukee, WI) took three measurements, each 1 min apart. At baseline and at the end of each intervention period, fasting (12 ± 2 h, water only) blood draws were collected for measurements of glucose, insulin, high-sensitivity C-reactive protein (hs-CRP), lipoprotein lipids, and lipoprotein subfraction cholesterol and particle concentrations; a short, 40-min intravenous glucose tolerance test (IVGTT) was also performed at baseline and on the final day of each intervention period.

Fasting glucose was assessed using an enzymatic colorimetric assay and fasting insulin was assessed using an electrochemiluminescence immunoassay (Cleveland HeartLab, Cleveland, OH). For the short IVGTT, an indwelling catheter was inserted to deliver the glucose load and obtain blood samples. To maintain patency of the IV catheter, normal saline solution was used to flush the catheter and infused as a slow, continuous drip. Blood samples were collected at *t* = −5, 5, 10, 20, 30, and 40 ± 1 min, where an IV glucose bolus [0.3 g/kg body weight, 50% dextrose solution (with a maximum of 25 g of glucose)] was provided over ~1.5 min at *t* = 0. The insulin sensitivity index (ISI) was calculated as the fractional glucose disappearance rate (Kg; calculated as −1 × the slope of the value for log_e_ glucose on time from 10–40 min) divided by the mean plasma insulin concentration from 10 to 40 min [i.e., total area under the curve (AUC)/30 min] [19, 20]. Acute insulin response to glucose (AIRg) was calculated as the average increment in glucose during the first 10 min after the glucose bolus. The disposition index (DI; ISI × AIRg), a measure of pancreatic beta-cell function, was also calculated. Fasting homeostasis model assessment of insulin sensitivity (HOMA2-%S), beta-cell function (HOMA2-%B), and insulin resistance (HOMA-IR) were assessed from fasting glucose and insulin values (www.dtu.ox.ac.uk/homacalculator/index.php).

Fasting total cholesterol (TC), high-density lipoprotein cholesterol (HDL-C), and TG concentrations were analyzed according to the Standardization Program of the Centers for Disease Control and Prevention and the National Heart, Lung and Blood Institute using enzymatic colorimetric methodology. Low-density lipoprotein cholesterol (LDL-C) concentrations (mg/dL) were calculated according to the Friedewald equation as follows in mg/dL: LDL-C = TC − HDL-C − TG/5. Non-HDL-C concentrations (mg/dL) were calculated as: Non-HDL-C = TC − HDL-C. Fasting lipoprotein subfraction concentrations and LDL particle concentration were analyzed using a density gradient ultracentrifugation technique, termed vertical auto profile (VAP Diagnostics Laboratory, Inc. Birmingham, AL) [21–24]. Fasting hs-CRP concentrations were assessed using an immunoturbidimetric assay (Cleveland HeartLab, Cleveland, OH) [25].

### Statistical analysis

Sample size calculations indicated that an evaluable sample of 30 subjects would provide 80% power to detect a difference of 0.54 standard deviations between diet conditions for the primary outcome variable, the ISI from the short IVGTT. A sample of 39 subjects was randomized to account for attrition. All tests of significance were performed at alpha = 0.05, two-sided. Analyses utilized data from all subjects who provided data during both diet conditions. Statistical comparisons were made for differences between conditions for changes or percent changes from baseline.

Statistical modeling for continuous variables was completed using Proc Mixed repeated measures analysis of variance or covariance (SAS for the personal computer, version 9.3, SAS Institute, Cary, NC). The initial models contained terms for diet condition, sequence, period, and baseline value as a covariate, with subject as a random effect. Models were reduced in a stepwise manner until only significant (*p* < 0.05) terms or diet condition and baseline remained in the model. Assumptions of normality of residuals were investigated for each model. If significant non-normality for a variable, or class of variables, was detected using the Shapiro–Wilk test, with an alpha level of 0.01, an analysis using rank transformation was applied [26]. Separate models were run to evaluate potential carryover effects (treatment by sequence interactions). None were evident; therefore, only pooled data from both sequence groups are presented. Possible differences between treatment conditions for dichotomous variables were assessed using McNemar’s test or a binomial test.

Sensitivity analyses were completed to investigate possible heterogeneity in responses for key outcome variables according to age subgroups (< or ≥ median age of 54.1 years) and in those with (*n* = 19) and without (*n* = 11) ≥3 metabolic syndrome criteria.

## Results

**Fig. 1:**
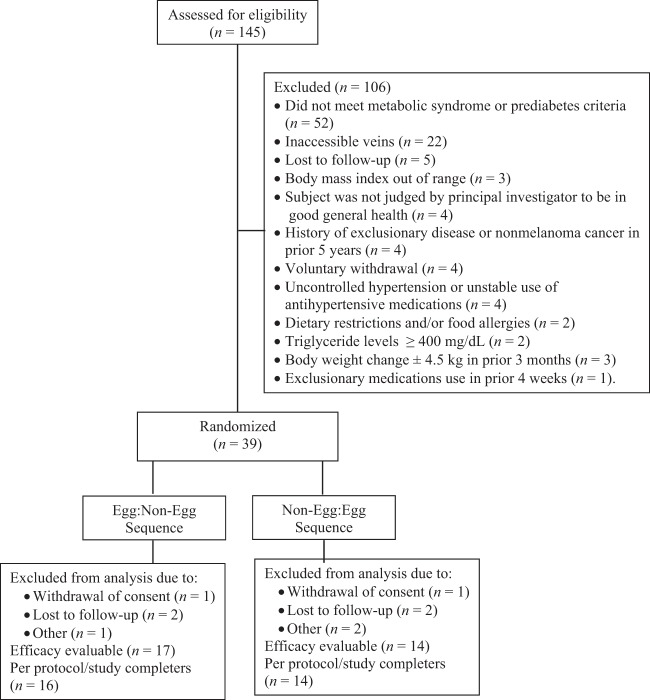
Flow diagram of subjects assessed for eligibility, excluded, randomized and analyzed for the study.

A total of 145 volunteers were screened, 39 eligible subjects were randomized to a diet condition sequence, and 30 (19 females and 11 males) completed both treatment conditions and were, therefore, included in the analysis sample (Fig. 1). The primary reason for screen failure was not meeting entry criteria for having metabolic syndrome or prediabetes (*n* = 52). Other reasons included not having accessible veins for blood draws (*n* = 22), lost to follow-up (*n* = 5), not meeting BMI criteria (*n* = 3), not meeting general health criteria as determined by the principal investigator based on medical history and/or screening laboratory results (*n* = 4), having a history of exclusionary disease (*n* = 4), voluntary withdrawal (*n* = 4), having uncontrolled hypertension or unstable use of antihypertensive medications (*n* = 4), having a significant change in body weight in the prior 3 months (*n* = 3), having dietary restrictions and/or food allergies (*n* = 2), having a TG level ≥400 mg/dL (*n* = 2) and using exclusionary medications in the 4 weeks prior to the study (*n* = 1). Nine subjects did not complete the trial due to withdrawal of consent (*n* = 5) or loss to follow-up (*n* = 4).

Baseline demographic and anthropometric characteristics and physical activity as well as baseline metabolic syndrome component and prediabetes characteristics are presented in Table 1. The mean nutrient compositions of the Egg and Non-Egg breakfast conditions differed in sugar and protein content (Supplementary Table). Specifically, the mean energy content of the 3-day rotating breakfasts for the Egg and Non-Egg conditions were 554 and 555 kcal, respectively. Mean sugar content was 25 g higher (13 g for Egg; 37 g for Non-Egg) in the Non-Egg breakfast condition, and mean protein content was 19 g higher (36 g for Egg; 17 g for Non-Egg) in the Egg breakfast condition. This resulted in 9% of energy from sugar and 27% of energy from protein in the Egg condition, and 27% of energy from sugar and 12% of energy from protein in the Non-Egg condition.

**Table 1:** Demographic and anthropometric baseline characteristics and metabolic syndrome components and the presence of prediabetes at baseline.

Characteristic	Study completers sample (*N* = 30)
	*n* (%)
Sex	
Male	11 (36.7)
Female	19 (63.3)
Race	
White/Caucasian	17 (56.7)
Black/African American	7 (23.3)
Other	6 (20.0)
Ethnicity	
Not Hispanic/Latino	21 (70.0)
Hispanic/Latino	6 (20.0)
Not reported	3 (10.0)
Current smoker	4 (13.3)
Consumes alcohol	21 (70.0)

	Mean (SEM) or *n* (%) or median (IQL)
Age (y)	54.1 (1.9)
Height (cm)	167 (1.6)
Weight (kg)	89.6 (2.4)
BMI (kg/m^2^)	31.9 (0.7)
BMI ≥ 25.0 to <30.0 kg/m^2^	13 (43.3)
BMI ≥ 30.0 kg/m^2^	17 (56.7)
Waist circumference (cm), men	100 (3.1)
Waist circumference (cm), women	102 (1.5)
Physical activity (MET hours/week)	240 (231, 275)

Metabolic syndrome components/prediabetes	
	*n* (%)
TG ≥ 150 mg/dL	13 (43.3)
HDL-C < 40 mg/dL, Men	3 (27.3)
HDL-C < 50 mg/dL, Women	10 (52.6)
SBP ≥ 130 or DBP ≥ 85 mm Hg or BP Medication	18 (60.0)
Men, waist circumference ≥102 cm	5 (45.5)
Women, waist circumference, ≥88 cm	19 (100)
Presence of prediabetes	
Fasting glucose 100–125 mg/dL	16 (53.3)
HbA1c 5.7–6.4%	14 (46.7)
Number of metabolic syndrome components	
1	5 (16.7)
2	6 (20.0)
3	11 (36.7)
4	6 (20.0)
5	2 (6.7)
≥3	19 (63.3)

BMI subgroups are *n* (%), physical activity MET hours/week are Median (IQL), and others in this section are Mean (SEM).

Metabolic syndrome component criteria differed by sex for HDL-C and waist circumference; thus, n values presented were derived from an eligible total of *n* = 11 men and *n* = 19 women. The adjacent percentages represent the percentage of the total subject population.

*BMI* body mass index, *BP* blood pressure, *DBP* diastolic blood pressure, *HbA1c* glycated hemoglobin, *HDL-C* high-density lipoprotein cholesterol, *IQL* interquartile limits, *MET* metabolic equivalent, *SBP* systolic blood pressure, *SEM* standard error of the mean, *TG* triglycerides.

Median daily energy and nutrient intakes at baseline and during each diet condition are presented in Table 2. Based on daily logs, breakfast accounted for ~25% of the total energy intake during each condition. Participants had a median compliance with study food intake of 100% [interquartile limits (IQL) 97.0, 100] during both the Egg intervention and the Non-Egg intervention (median 100%; IQL 97.0, 103). There was no significant change from baseline in physical activity, measured as metabolic equivalent (MET) hours/week, during either the Egg or Non-Egg condition, and no significant difference in changes from baseline between conditions.

**Table 2:** Median daily energy, macronutrient, and select nutrient intakes at baseline and during Egg and Non-Egg conditions in the study completers (*N* = 30).

	Median (IQL)			
Parameter	Baseline	Egg	Non-Egg	*P* value
Energy, kcal/day	2111 (1512, 2437)	2145 (1820, 2626)	1996 (1592, 2285)	**0.008**
CHO, % energy	44.8 (39.7, 55.3)	46.6 (38.3, 51.0)	48.7 (45.2, 51.0)	**0.013**
Sugar, % energy	14.7 (11.2, 18.2)	16.3 (11.5, 20.1)	17.1 (14.4, 21.2)	0.169
Protein, % energy	18.0 (14.7, 24.2)	18.3 (15.9, 21.4)	17.4 (15.1, 20.6)	0.108
Total fat, % energy	38.2 (29.8, 40.6)	35.0 (32.4, 37.7)	34.8 (33.7, 37.5)	0.565
SFA, % of energy	11.9 (8.6, 13.0)	11.8 (10.2, 12.8)	11.2 (9.3, 11.6)	0.388
UFA, % of energy	25.5 (20.4, 28.1)	23.8 (20.4, 26.4)	23.6 (22.0, 25.7)	0.867
Dietary fiber, g/day	17.0 (12.3, 25.8)	18.4 (15.5, 23.7)	16.5 (14.8, 19.7)	0.059
Cholesterol, mg/day	318 (199, 441)	550 (501, 633)*	190 (140, 293)*	**<0.001**
Sodium, mg/day	3077 (2356, 4404)	3827 (3194, 4556)*	3369 (2625, 3717)	**0.011**
Calcium, mg/day	596 (479, 926)	600 (493, 671)	739 (596, 800)	0.066

Values for UFA are shown instead of separately as monounsaturated fatty acids (MUFA) and polyunsaturated fatty acids (PUFA), because the Food Processor® Nutrition Analysis software utilizes only the information contained in the Nutrition Facts label (i.e., total fat, saturated fat, and trans fat), which, therefore, allows calculation of UFA, but calculations of MUFA and PUFA would be inaccurate.

*CHO* carbohydrates, *IQL* interquartile limits, *SFA* saturated fatty acids, *UFA* unsaturated fatty acids.

*P* values are for the comparison of treatment conditions in the change from baseline.

Bold values indicates statistical significant *p*-values.

An asterisk (*) denotes that the change from baseline was statistically significant, *p* < 0.05.

Reported median (IQL) daily energy intake during the Egg condition was 2145 kcal (1820, 2626), which was significantly (*p* = 0.008) higher than the 1996 kcal (1592, 2285) reported during the Non-Egg condition. During the Egg condition, 73.3% [95% confidence interval (CI) 57.5–89.2%; *p* = 0.008] of the subjects had higher energy intake than during the Non-Egg condition. Diet record analyses indicated the 149-kcal/day difference was due to intake of foods other than the study products. Despite the higher reported energy intake during the Egg condition, weight change from baseline did not differ between the interventions: −0.1 ± 0.3 kg and 0.2 ± 0.3 kg, for the Egg and Non-Egg conditions, respectively. Median (IQL) percent energy from CHO was significantly (*p* = 0.013) higher during the Non-Egg condition [48.7% (45.2, 51.0)] compared with the Egg condition [46.6% (38.3, 51.0)]. Median daily sodium intake during the Egg condition was 3827 mg (3194, 4556), which was significantly (*p* = 0.011) higher than the 3369 mg (2625, 3717) during the Non-Egg condition, and significantly (*p* < 0.05) higher than sodium intake at baseline [3077 mg (2356, 4404)]. This difference was mainly attributable to differing sodium contents of the study foods. Median daily cholesterol intake during the Egg condition was 550 mg (501, 633), which was significantly (*p* < 0.001) higher than the 190 mg (140, 293) during the Non-Egg condition, and significantly (*p* < 0.05) higher than cholesterol intake at baseline [318 mg (199, 441)].

Neither intake of the Egg nor Non-Egg breakfasts significantly altered the primary outcome variable, ISI (Table 3), nor were there significant differences between or within diet conditions observed for DI, Kg, AIRg, and AUC_0–10min_, AUC_0–40min_, and AUC_10–40min_ for insulin. Fasting glucose and insulin and fasting HOMA2-%S and HOMA2-%B indices were also not significantly affected by diet condition. However, the Non-Egg condition significantly (*p* = 0.028) increased HOMA-IR compared with the Egg condition (24.4 ± 13.1 vs. 1.4 ± 9.9%, respectively) (Table 3).

**Table 3:** Short intravenous glucose tolerance test outcomes and carbohydrate metabolism parameters at baseline, end of treatment and percent changes from baseline following Egg and Non-Egg conditions in the study completers (*N* = 30).

	Mean (SEM) or median (IQL)					
Parameter	Baseline	Egg, EOT	Non-Egg, EOT	Egg, % ∆	Non-Egg, % ∆	*P* value
ISI × 10^−4^ min^−1^ (mU/L)^−1^	8.9 (2.7)	6.8 (0.9)	5.4 (0.9)	15.0 (14.3)	−4.1 (16.8)	0.193
AIRg, mU/L	28.2 (19.4, 49.0)	22.4 (13.8, 55.9)	34.6 (17.0, 55.2)	−18.7 (−39.5, 14.2)	3.6 (−35.3, 43.7)	0.234
DI × 10^−4^	156 (71.1, 324)	158 (40.1, 296)	143 (48.6, 289)	−17.3 (−47.2, 33.6)	−18.5 (−63.2, 13.2)	0.579
Kg, %	1.6 (1.1, 2.9)	1.4 (1.2, 2.1)	1.4 (0.9, 2.0)	−1.5 (−31.8, 48.1)	−6.6 (−33.9, 23.0)	0.213
Insulin AUC_0–10min_	406 (265, 647)	416 (220, 720)	499 (299, 679)	−12.1 (−37.7, 25.2)	5.4 (−12.5, 32.9)	0.110
Insulin AUC_10–40min_	1010 (591, 1868)	932 (728, 1642)	1046 (620, 2160)	2.4 (−15.2, 20.2)	1.9 (−24.0, 18.0)	0.808
Insulin AUC_0–40min_	1409 (928, 2736)	1333 (851, 2740)	1582 (962, 2951)	1.4 (−18.9, 14.1)	−0.6 (−23.6, 17.7)	0.708
Fasting glucose, mg/dL	90.3 (2.7)	90.8 (2.0)	92.3 (1.9)	2.3 (2.8)	4.1 (3.0)	0.459
Fasting insulin, mU/L	9.5 (6.1, 12.1)	8.2 (5.9, 14.5)	9.0 (6.1, 15.3)	−0.6 (−23.7, 10.7)	8.6 (−14.0, 28.1)	0.057
HOMA2%-B	101 (83.1, 166)	89.5 (66.8, 127)	104 (72.7, 170)	−2.0 (−23.3, 7.4)	−0.6 (−15.6, 15.3)	0.277
HOMA2%-S	86.8 (66.4, 122)	95.0 (55.0, 127)	85.3 (49.5, 123)	1.9 (−9.0, 29.1)	−8.5 (−25.2, 15.0)	0.067
HOMA-IR	2.5 (0.3)	2.3 (0.3)	2.7 (0.3)	1.4 (9.9)	24.4 (13.1)	**0.028**

Units for insulin AUCs are mU/L × min.

*% ∆* percent change, *AIRg* acute insulin response to glucose, *AUC* area under the curve, *DI* disposition index, *EOT* end of treatment, *HOMA2%-B* homeostasis model assessment 2-beta-cell function, *HOMA2-%S* homeostasis model assessment 2-insulin sensitivity, *HOMA-IR* homeostasis model assessment 2-of insulin resistance, *IQL* interquartile limits, *ISI* insulin sensitivity index, *Kg* fractional disappearance of glucose constant, *SEM* standard error of the mean.

Bold value indicate statistical significant *p*-value.

*P* values are for the comparison of treatment conditions in the percent change from baseline.

Median (IQL) LDL-C decreased (*p* < 0.05) by 6.0% (−11.7, 2.9) from a baseline concentration of 119 mg/dL (84.5, 139) after 4-week intake of the Non-Egg breakfast foods, which was larger than the 2.9% (−6.0, 6.2) reduction during the Egg condition (*p* = 0.023 between diets) (Table 4). The 4-week intake of the Egg breakfasts resulted in a numerically larger reduction in mean ± standard error of the mean (SEM) HDL-C of 0.6 ± 1.7% from a baseline of 54.7 ± 2.3 mg/dL, but this reduction did not differ significantly from the change in HDL-C levels following the Non-Egg breakfast intervention. Mean ± SEM systolic blood pressure was reduced more during the Egg condition versus the Non-Egg condition (−2.7 ± 1.1 vs. 0.0 ± 1.4%, respectively, *p* = 0.018) from a baseline value of 127 ± 2.1 mm Hg. Neither intake of the Egg breakfasts nor the Non-Egg breakfasts significantly affected hs-CRP levels.

**Table 4:** Fasting lipoprotein lipids, high-sensitivity C-reactive protein (hs-CRP) and blood pressures at baseline, end of treatment and percent changes from baseline following Egg and Non-Egg conditions in the study completers (*N* = 30).

	Mean (SEM) or median (IQL)					
Parameter	Baseline	Egg, EOT	Non-Egg, EOT	Egg, % ∆	Non-Egg, % ∆	*P* value
TC, mg/dL	195 (8.1)	194 (9.3)	189 (7.6)	−0.8 (1.4)	−2.4 (1.6)	0.353
LDL-C, mg/dL	119 (84.5, 139)	113 (86.5, 141)	106 (88.5, 132)	−2.9 (−6.0, 6.2)	−6.0 (−11.7, 2.9)*	**0.023**
HDL-C, mg/dL	54.7 (2.3)	54.1 (2.4)	54.6 (2.7)	−0.6 (1.7)	−0.1 (1.9)	0.776
Non-HDL-C, mg/dL	141 (7.5)	140 (8.7)	135 (7.1)	−1.1 (1.9)	−3.4 (1.9)	0.271
TG, mg/dL	133 (72.0, 150)	111 (64.0, 176)	117 (64.0, 179)	0.7 (−12.7, 17.9)	−0.6 (−12.4, 10.1)	0.584
TC:HDL-C	3.7 (0.2)	3.7 (0.2)	3.6 (0.2)	0.04 (1.7)	−1.8 (1.7)	0.280
hs-CRP, mg/L	1.4 (0.8, 3.9)	2.0 (0.7, 3.5)	1.5 (0.8, 3.9)	−11.5 (−38.1, 17.4)	0.0 (−28.2, 31.3)	0.349
SBP, mm Hg	127 (2.1)	123 (2.2)	126 (2.5)	−2.7 (1.1)*	0.0 (1.4)	**0.018**
DBP, mm Hg	81 (1.9)	79 (1.3)	79 (1.4)	−1.9 (1.7)	−0.7 (2.1)	0.351

*% ∆* percent change, *DBP* diastolic blood pressure, *EOT* end of treatment, *HDL-C* high-density lipoprotein cholesterol, *hs-CRP* high-sensitivity C-reactive protein, *IQL* interquartile limits, *LDL-C* low-density lipoprotein cholesterol, *Non-HDL-C* non-high-density lipoprotein cholesterol, *SBP* systolic blood pressure, *SEM* standard error of the mean, *TC* total cholesterol, *TG* triglycerides.

*P* values are for the comparison of treatment conditions in the percent change from baseline.

Bold values indicates statistical significant *p*-values.

An asterisk (*) denotes that the percent change from baseline was statistically significant, *p* < 0.05.

VAP assessment of lipoprotein subfraction cholesterol concentrations and LDL particles indicated no significant effects of either of the dietary interventions on these parameters (Table 5).

**Table 5:** Fasting lipoprotein subfractions and particles assessed by vertical auto profile methodology at baseline, end of treatment and changes from baseline following Egg and Non-Egg conditions in the study completers (*N* = 30).

	Mean (SEM) or median (IQL)					
Parameter	Baseline	Egg, EOT	Non-Egg, EOT	Egg, ∆	Non-Egg, ∆	*P* value
LDL_1+2_-C, mg/dL	32.2 (3.3)	34.5 (3.2)	32.0 (2.5)	2.3 (2.1)	−0.3 (1.6)	0.088
LDL_3+4_-C, mg/dL	52.1 (4.2)	54.8 (4.2)	55.6 (4.1)	2.7 (3.2)	3.6 (3.8)	0.673
HDL_2_-C, mg/dL	12.5 (9.0, 17.0)	12.0 (9.0, 17.0)	12.0 (9.0, 18.0)	0.0 (−2.0, 1.0)	0.0 (−1.0, 1.0)	0.665
HDL_3_-C, mg/dL	37.1 (1.9)	38.3 (1.3)	38.9 (1.4)	1.1 (1.6)	1.8 (1.4)	0.350
LDL particle, nmol/L	1484 (1083, 2086)	1540 (1156, 1803)	1311 (1068, 1774)	1.0 (−182, 275)	−124 (−306, 117)	0.117
LDL max time, sec	115 (0.5)	115 (0.5)	115 (0.5)	0.0 (0.3)	−0.2 (0.3)	0.380

*∆* change, *EOT* end of treatment, *HDL* high-density lipoprotein, *IQL* interquartile limits, *LDL* low-density lipoprotein, *SEM* standard error of the mean.

*P* values are for the comparison of treatment conditions in the change from baseline.

Sensitivity analyses showed no statistically significant, or clinically material, differences in key outcome variables according to age or metabolic syndrome criteria (data not shown).

## Discussion

The results of this randomized crossover study show that a 4-week substitution of higher-CHO, Non-Egg breakfast foods with Egg-based breakfast foods had modest effects on the cardiometabolic risk-factor profile in men and women at risk for T2D. There were no significant differences observed for the primary outcome variable, ISI determined from the short, 40-min IVGTT. However, HOMA-IR was significantly increased following the Non-Egg (24.4%) compared with the Egg condition (1.4%). Although this finding suggests that replacing higher-CHO (primarily sugar) foods with egg-based foods at breakfast may have a favorable effect on whole-body insulin sensitivity, caution is warranted. The HOMA-IR value is calculated using a linear model based on population-derived estimates, whereas HOMA2-%S is calculated using a nonlinear model, which is theoretically more robust [19, 20, 27]. No significant differences were present between the Egg and Non-Egg conditions for HOMA2-%S based on fasting values, or the ISI from the short IVGTT. Also, because there were several comparisons conducted, the possibility of a type I statistical error cannot be ruled out. Regardless, all three assessments showed directionally similar results and indicated no adverse effect on whole-body insulin sensitivity of Egg intake compared with the habitual diet at baseline, or compared with the Non-Egg condition.

Prior research from our group showed that partial replacement of CHO with a combination of UFA and egg protein resulted in a net 24% increase in insulin sensitivity [12]. Results from another study completed by our group [28] also showed favorable effects on insulin sensitivity with the replacement of dietary CHO. Daily consumption of three servings of sugar-sweetened products reduced insulin sensitivity by 18% as assessed by HOMA2-%S, compared with a habitual diet at baseline, whereas three servings of dairy products produced no change.

The results of the present trial are consistent with a neutral to modestly favorable effect on whole-body insulin sensitivity of replacing higher-CHO, non-egg-based foods at the breakfast meal with whole eggs, which are higher in protein. This aligns with results reported by Gadgil et al., where the partial replacement of CHO with UFA resulted in a significant (*p* < 0.05) increase in fasting indicators of insulin sensitivity, whereas replacing CHO with protein produced a smaller and nonsignificant increase [29]. Furthermore, Chiu et al. reported that high (30% of energy) and moderate (20% of energy) protein diets did not significantly affect ISI or the DI assessed with minimal model analysis of data from IVGTT in overweight or obese adults, as compared with a lower protein (15% of energy) control diet that was higher in CHO (55 vs. 35% of energy) [30]. One possible explanation for the present results compared with those from our prior investigations may be that egg protein intake has minimal, or no effect on insulin sensitivity and that the increase in insulin sensitivity observed in our prior study was driven mainly by the effect of higher UFA intake. Another possibility is that the quality of CHO affects insulin sensitivity. Our two prior studies replaced a combination of added sugars and refined starches with energy from egg protein and UFA or from dairy foods, whereas the present study primarily replaced sugar with energy from whole eggs [12, 28].

Another potentially relevant factor regarding effects of different meals on CHO metabolism is time of day. Differences in sympathetic nervous system activity and/or diurnal patterns related to the release of incretin hormones (e.g., glucagon-like peptide-1 and gastric inhibitory polypeptide in response to a meal) may affect insulin sensitivity [31], and markedly higher (~40%) insulin sensitivity has been observed in the morning compared with mid-afternoon or evening [32]. Jakubowicz et al. conducted a randomized crossover trial where subjects with T2D were fed either a meal pattern that included a high-energy breakfast plus a low-energy dinner (breakfast: 2946 kJ, lunch: 2523 kJ, and dinner: 858 kJ) or a meal pattern with a low-energy breakfast plus a high-energy dinner (breakfast: 858 kJ, lunch: 2523 kJ, and dinner: 2946 kJ) [32]. Despite isoenergetic intakes, those consuming the higher energy breakfast meal pattern had reduced postprandial hyperglycemia and higher levels of intact and total glucagon-like peptide-1. In the present trial, study products were consumed at the breakfast meal, when insulin sensitivity would be expected to be at its highest. It is uncertain whether similar results would be obtained with consumption of the study products in the afternoon or evening.

Another contributing factor to the observed results may be that the breakfast meal accounted for ~25% of the total energy intake during each condition and thus subjects may have partially compensated for differences in composition of breakfast meals during the remainder of the day. The differing nutrient compositions of the breakfasts during the treatment conditions yielded daily differences in total energy, total CHO energy, dietary cholesterol and sodium, but did not translate into daily dietary differences in percentage of energy from sugars or protein. The Egg and Non-Egg breakfasts differed in CHO and sugar contents, as well as glycemic load, which can affect glucose metabolism [33]. As shown in Table 2, total daily differences in CHO and sugar intakes were small. Results may have been different had the breakfast meals differed more markedly in CHO content and glycemic load, which may have resulted in larger differences in total daily intakes of these parameters. However, the objective was to compare the effects of commonly eaten egg-based and non-egg-based breakfast foods, reflecting “real world” eating patterns.

The subjects enrolled in this study were regular egg consumers, who were willing to participate in a study involving egg consumption and who, at baseline, had a dietary cholesterol intake slightly higher than the average intake in the U.S. population (318 mg/d vs. 278 mg/d) [34]. Consumption of 12 eggs/week contributes an average of ~315 mg/d of cholesterol to the diet. Compared with baseline, median dietary cholesterol intake, based on diet record analysis, was 128 mg/d lower during the Non-Egg condition and 232 mg/d higher during the Egg condition, a difference between conditions of 360 mg/d. The predicted effect of a reduction of 128 mg/d in dietary cholesterol, based on our previously published meta-regression analysis of studies in which dietary cholesterol was modified, is a 5.5 mg/dL reduction in LDL-C [35], which is similar to the observed 6.5 mg/dL LDL-C reduction observed in the Non-Egg condition. The predicted effect of a 232-mg/day increase in dietary cholesterol intake is a 7.4 mg/dL increase in LDL-C; however, we observed a 3.5 mg/dL reduction in LDL-C in the Egg condition. The small, nonsignificant differences in intakes of saturated fat and UFA from diet record analyses cannot explain the differences in LDL-C responses [36].

Results from previous studies in which egg consumption has been increased have shown mixed results, with some showing increases in LDL-C and others showing neutral effects or a modest decline in LDL-C [37]. In our previous meta-regression analysis of studies that evaluated the effects of changes in dietary cholesterol on LDL-C level [35], we noted that the increase in LDL-C was ~50% lower when cholesterol was provided via egg yolks than with other forms (unpublished results, not tested statistically). Thus, it is possible that some component or components of egg yolks influence the LDL-C response, e.g., by interfering with cholesterol absorption or altering hepatic cholesterol handling. For example, results from previous research indicate that higher intake of phospholipids can inhibit cholesterol absorption in vitro, in animal studies and in limited studies in humans [38]. Egg yolks have one of the highest levels of dietary phospholipids (about 1.75 g total phospholipids per yolk) [38, 39], and this may explain, at least in part, the lower than expected circulating cholesterol response to higher dietary cholesterol intake during the Egg condition. It is also possible that changes in dietary intake at eating occasions other than breakfast affected LDL-C levels. Additional research will be needed to confirm this finding and to evaluate potential mechanistic explanations.

Results from a recent pooled analysis of data from six US prospective cohort studies (29,615 participants) indicated that both higher dietary cholesterol intake and higher egg intake were associated with higher incident CVD and all-cause mortality over a median follow-up period of 17.5 years [40]. In the analysis, median cholesterol intake was 241 mg/d (IQR 164, 350) and median egg intake was 0.14 eggs/day (IQL 0.07, 0.43). In multivariate models that adjusted for intakes of nutrients and diet quality, for every 0.5 egg/day increment in intake, there was a 6% higher rate of incident CVD and a 7% higher rate of all-cause mortality. Both associations lost significance after adjustments for dietary cholesterol, suggesting that the cholesterol content of eggs is responsible for the associations [40]. Notably, adjustment for CVD risk markers, including non-HDL-C and HDL-C, did not materially alter the point estimates for the associations of egg or cholesterol intakes with incident CVD or mortality.

These results from US cohorts differ from those from the China Kadoorie Biobank cohort of 461,213 participants that had a median follow-up period of 8.9 years [41]. At baseline, 13.1% of subjects reported daily consumption of eggs (average of 0.76 eggs/day) and these subjects showed 11% lower risk for incident CVD [hazard ratio (HR) 0.89, 95% CI 0.87–0.92] than those who consumed eggs rarely or never. Daily egg consumers also showed 10–18% lower incidence rates for ischemic heart disease, stroke (ischemic and hemorrhagic) and CVD death. In multivariate analyses, each 1 egg/week increase in consumption was associated with a 3% lower risk for incident CVD (HR 0.97, 95% CI 0.96–0.98).

Other large, prospective cohort studies in the USA and elsewhere have shown conflicting results regarding effects of dietary cholesterol and/or egg intake on incident CVD [4, 39, 42–46]. Thus, at present, the impact of consuming eggs and dietary cholesterol on CVD risk is uncertain. The impact of consuming 12 eggs/week on mean LDL-C level was modest in the present study, and the authors view these results as consistent with the view that eggs can be included in a healthy dietary pattern. A recent Science Advisory from the American Heart Association recommended that healthy individuals can include up to one whole egg or equivalent daily as part of a healthy dietary pattern [47].

Diet record analyses indicated a statistically significant 149 kcal/day higher intake of energy from non-study foods during the Egg condition versus the Non-Egg condition, and this higher energy intake was observed in 73% of subjects. However, despite this, weight change from baseline did not differ between the interventions (−0.1 and 0.2 kg, for Egg and Non-Egg conditions, respectively). A 149 kcal/day difference over a 4-week period would be expected to yield roughly 0.5 kg higher weight for the Egg condition. Instead, net body weight was nonsignificantly lower (−0.3 kg) during the Egg condition versus the Non-Egg condition. Results from a trial by Ebbeling et al. showed that after a 12-week weight loss run-in, subjects assigned to a low-CHO diet (20% of daily energy) versus a high-CHO diet (60% of daily energy) for 20 weeks had a 91 kcal/day greater total energy expenditure [48]. Total energy expenditure differed by diet with a linear trend of ~52 kcal/day for every 10% decrease in the contribution of CHO calories (as a replacement for dietary fat). In the present study, CHO intake was lower during the Egg condition by ~2% of energy compared with the Non-Egg condition and the difference was primarily due to higher protein intake during the Egg condition. Protein has been demonstrated to produce a higher thermic effect of food compared with CHO [49], which may have contributed to a need to consume more energy to maintain body weight. Physical activity questionnaire calculations suggested that subjects maintained habitual physical activity during both intervention periods, although the sensitivity of such assessments is low [50]. While acknowledging the limitations inherent in estimating energy intake from diet records, these results suggest that the effects of egg consumption on the determinants of body weight regulation (i.e., energy expenditure and appetite) should be further explored.

Despite a significant (*p* < 0.05) 750 mg/d increase from baseline sodium intake during the Egg condition, mean systolic blood pressure was significantly (*p* < 0.05) reduced by 2.7% from baseline during the Egg condition (*p* = 0.018 compared with the Non-Egg condition). Increased sodium intake without an accompanying increase in blood pressure with egg protein intake has been observed previously by our group [12]. An inverse association of egg intake with risk for hypertension has also been noted in some observational research [51]. For example, a 3-year prospective study from Iran found that those with the highest tertile of egg intake (23.3 g/day) had a 46% significant reduction in hypertension occurrence versus those in the lowest tertile (6.4 g/day); no other associations with blood pressure were observed for any other protein sources [51]. However, others have failed to observe any effect of egg intake on blood pressure [52]; thus, more research is needed to better understand the effects of egg consumption on blood pressure regulation.

Strengths of this study include the use of commonly consumed foods for both diet conditions and the incorporation of these foods into a habitual diet. Since both design aspects mimic commonplace situations, the results are likely to be generalizable. A limitation of the trial is the reliance on dietary reporting by the subjects. Prior studies have shown that, on average, individuals under-report energy intake by 20% [53], with even greater underreporting among subjects with obesity [54]. Thus, we cannot rule out the possibility of systematic errors in dietary intake data provided by subjects, which could produce bias. Another limitation is the relatively short intervention period of 4 weeks. It will be of interest to assess whether adaptation occurs over longer periods. Also of interest would be an investigation into the effects of egg intake according to the number of metabolic syndrome criteria, which could not be meaningfully assessed in the present trial due to the relatively small sample size.

## Conclusion

Intake of egg-based breakfast meals (12 eggs/week), compared with consumption of non-egg, higher-CHO breakfast meals, did not adversely affect insulin sensitivity and other aspects of CHO homeostasis. LDL-C declined less from baseline (−2.9%) during the Egg condition than during the Non-Egg condition (−6.0%), while systolic blood pressure was reduced by 2.7% during the Egg condition and was unchanged during the Non-Egg condition. HOMA-IR was lower by a net difference of 23% during the Egg condition than the Non-Egg condition, but smaller and nonsignificant differences were observed for other indices of insulin sensitivity. No other significant differences were observed in the cardiometabolic risk-factor profile. Of interest, egg intake at the breakfast meal was associated with increased reported daily energy intake from non-study foods but was not associated with increased body weight. Additional research appears warranted to confirm this finding and investigate mechanistic explanations.

## Supplementary information

Supplementary Data Table
