# Supplementary material for: Effects of substituting eggs for high-carbohydrate breakfast foods on the cardiometabolic risk-factor profile in adults at risk for type 2 diabetes mellitus
Source: Eur J Clin Nutr. 2020 Mar 9;74(5):784–95. doi: 10.1038/s41430-020-0599-2 (PMC7214271; doi:10.1038/s41430-020-0599-2)
Supplement: Supplementary file 1 — Supplementary Data Table [file 41430_2020_599_MOESM1_ESM.docx]

**Supplementary Data Table.** Average energy and select nutrients provided by each 3-d rotating menu for the Egg breakfasts and Non-Egg breakfasts during each treatment condition.^1^

| **Parameter** | **Egg Study Foods** | | | | | **Non-Egg Study Foods** | | | | |
| --- | --- | --- | --- | --- | --- | --- | --- | --- | --- | --- |
|  | Taco Breakfast Scramble (eggs, potatoes, peppers, tomato, onions & cheese) | | Egg Breakfast Sandwich (eggs, cheese, ham & mustard on plain bagel) | Breakfast Burrito (tortilla-wrapped eggs, chicken strips, peppers, onions & BBQ sauce) | | Corn flakes RTE cereal with milk, yogurt-covered raisins, almonds & applesauce | Puffed rice RTE cereal with milk, granola bar, fruit cups & cheese | | Waffles with syrup, mixed dried fruit & cheese | |
| Calories (kcal) | 553 | 557 | | | 551 | 553 | | 558 | | 554 |
| Total Fat (g) | 19.8 | 20.9 | | | 17.6 | 20.8 | | 16.1 | | 19.9 |
| *SFA (g)* | 7.7 | 6.6 | | | 5.1 | 8.6 | | 5.7 | | 5.1 |
| *UFA (g)* | 12.1 | 14.3 | | | 12.5 | 12.2 | | 10.4 | | 14.8 |
| Total CHO (g) | 66.4 | 51.0 | | | 54.9 | 81.6 | | 84.1 | | 85.0 |
| *Sugars (g)* | 11.4 | 9.7 | | | 16.4 | 40.4 | | 36.2 | | 35.2 |
| *Fiber (g)* | 6.6 | 2.0 | | | 6.1 | 5.3 | | 4.1 | | 7.3 |
| Total Protein (g) | 28.0 | 38.2 | | | 41.1 | 14.9 | | 22.5 | | 12.9 |
| Cholesterol (mg) | 358 | 361 | | | 383 | 25.3 | | 29.5 | | 5.0 |

^1^Abbreviations: BBQ, barbeque; CHO, carbohydrate; RTE, ready to eat; SFA, saturated fatty acids; UFA, unsaturated fatty acids.
